# In-Plane Optical Beam Collimation Using a Three-Dimensional Curved MEMS Mirror †

**DOI:** 10.3390/mi8050134

**Published:** 2017-04-25

**Authors:** Yasser M. Sabry, Diaa Khalil, Bassam Saadany, Tarik Bourouina

**Affiliations:** 1 Department of Electronics and Communication Engineering, Faculty of Engineering, Ain-Shams University, 1 Elsarayat St., Abbassia 11517, Egypt; diaa_khalil@eng.asu.edu.eg; 2 Si-Ware Systems, 3 Khaled Ibn El-Waleed Street, Heliopolis, Cairo 11361, Egypt; bassam.saadany@si-ware.com (B.S.); tarik.bourouina@esiee.fr (T.B.); 3 Paris-Est, Laboratoire ESYCOM, ESIEE Paris, Cité Descartes, F-93162 Noisy-le-Grand CEDEX, France

**Keywords:** curved micromirrors, three-dimensional fabrication, Gaussian beams, surface roughness

## Abstract

The collimation of free-space light propagating in-plane with respect to the substrate is an important performance factor in optical microelectromechanical systems (MEMS). This is usually carried out by integrating micro lenses into the system, which increases the cost of fabrication/assembly in addition to limiting the wavelength working range of the system imposed by the dispersion characteristic of the lenses. In this work we demonstrate optical fiber light collimation using a silicon micromachined three-dimensional curved mirror. Sensitivity to micromachining and fiber alignment tolerance is shown to be low enough by restricting the ratio between the mirror focal length and the optical beam Rayleigh range below 5. The three-dimensional curvature of the mirror is designed to be astigmatic and controlled by a process combining deep, reactive ion etching and isotropic etching of silicon. The effect of the micromachining surface roughness on the collimated beam profile is investigated using a Fourier optics approach for different values of root-mean-squared (RMS) roughness and correlation length. The isotropic etching step of the structure is characterized and optimized for the optical-grade surface requirement. The experimental optical results show a beam-waist ratio of about 4.25 and a corresponding 12-dB improvement in diffraction loss, in good agreement with theory. This type of micromirror can be monolithically integrated into lensless microoptoelectromechanical systems (MOEMS), improving their performance in many different applications.

## 1. Introduction

**Figure 1:**
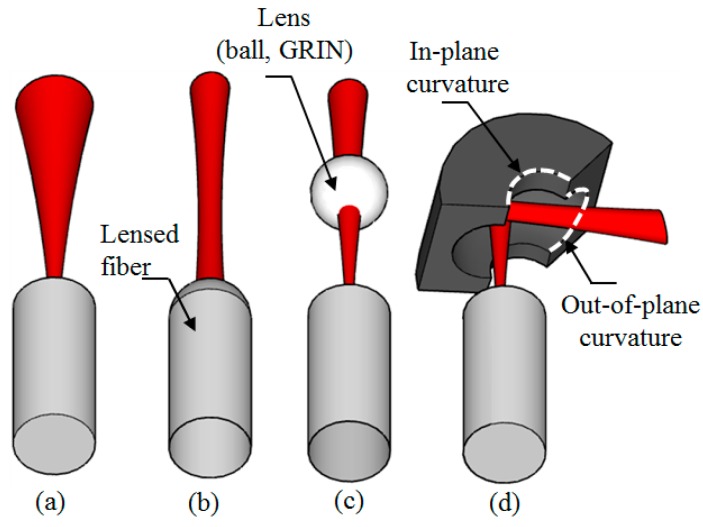
Optical beam propagation for the different architectures of (**a**) a cleaved fiber; (**b**) an integrated lens fiber; (**c**) an external lens; and (**d**) the proposed solution in this work.

Optical microelectromechanical systems (MEMS) technology has attracted great attention over the past couple of decades because of its reduced size, light weight and low cost [1]. There are two main architectures in the optical MEMS, namely in-plane architecture [2], where the light propagates from one component to another parallel to the substrate, and out-of-plane architecture [3], where the light hits the optical component either perpendicular to or with inclination on the substrate. For many applications, such as in optical telecommunication [1], optical coherence tomography [4] and on-chip sensing [5], the light source is connected to the optical MEMS device through a single-mode optical fiber, where the optical beam output from the fiber behaves as a Gaussian beam [2]. In this case, the propagation can be associated with beam size expansion before detection, leading to optical losses. This is even more serious in optical MEMS due to the size limit of the optical components [6,7]. Several solutions were introduced as shown in Figure 1 to overcome this challenge, such as the use of a lensed fiber [4] or an external lens integrated into the system in the form of a graded-index (GRIN) lens or a ball lens [6,7,8,9,10,11]. The lensed fiber solution is costly due to the piece-by-piece process of lens formation on the fibers, in addition to the reliability issue to possible fiber tip breakage. The external lens solution suffers from the cost and complexity of the assembly. In addition, refractive lenses have chromatic aberration and require anti-reflective coating to eliminate the reflection. The aberration and the coating both lead to limited working wavelength range.

Reflecting curved micromirrors are achromatic and can provide much a wider spectral response, but they need special attention during fabrication to obtain the curved surface. The common non-planar micro surfaces fabrication techniques are gray-tone mask [12], excimer laser [13], Reactive Ion Etching (RIE) lag effect [14] and photo resist (PR) reflow [15,16]. On one hand, non-silicon curved micromirrors were reported using a polymer dispensing and sucking technique [17], residual internal material stress resulting from deposition of gold on polysilicon for the purpose of light focusing [18], trapping of gas bubbles during melting a stack of small borosilicate glass tubes under a nitrogen atmosphere and further grinding and polishing for atomic studies [19] and deep silicon etching and PR reflow targeting optical interconnects [20]. On the other hand, silicon curved micromirrors fabricated on the wafer top surface were reported using isotropic chemical etching for the sake of optical detection of single atom [21], selective polishing method on the top of MEMS tunable vertical-cavity surface-emitting laser [22] and ion beam irradiation and electrochemical etching for atomic studies as well as optical interconnects [23]. The principal axis of the aforementioned micromirrors is oriented out-of-plane with the respect to the wafer substrate. This rendered the micromirror incompatible with silicon micro-optical bench systems where the light is propagating in-plane with respect to the substrate. Three-dimensional (3-D) micro optical bench systems requiring further assembly or mounting steps after fabrication were introduced in the literature. The most common is to use rotational assembly to create micro-optical subsystems that process free-space beams travelling above the surface of the chip [24]. Non-monolithically integrated mechanical mounting systems for connecting and aligning optical components on a micro optical bench (OB) were also reported [25,26]. This is, however, not compatible with the monolithic integration efforts for the microoptoelectromechanical systems (MOEMS) [27,28,29,30].

In this work, we demonstrate optical beam collimation and propagation loss reduction using a monolithic micromachined curved mirror with an in-plane principal axis, which is compatible with silicon micro-optical bench technology [31]. The paper is organized in the following manner. In Section 2, a theoretical study is carried out for the possibility of Gaussian beam collimation using curved surfaces exhibiting microscale focal lengths, i.e., not so large compared with the incident Gaussian beam Rayleigh range. The design of astigmatic micromirror curvatures is related to incidence angle of the incident Gaussian beam in order to generate a stigmatic collimated beam. The effect of the surface roughness of the micromirror is analyzed in Section 3. Then, the fabrication steps of the micromirror and the resulting structure are presented in Section 4. Finally, optical measurements are presented and discussed in Section 5 using the introduced curved micromirror for single-mode fiber output collimation and propagation loss reduction where the fiber axis lies in-plane with the substrate.

## 2. Theoretical Analysis of Optical Beam Collimation

**Figure 2:**
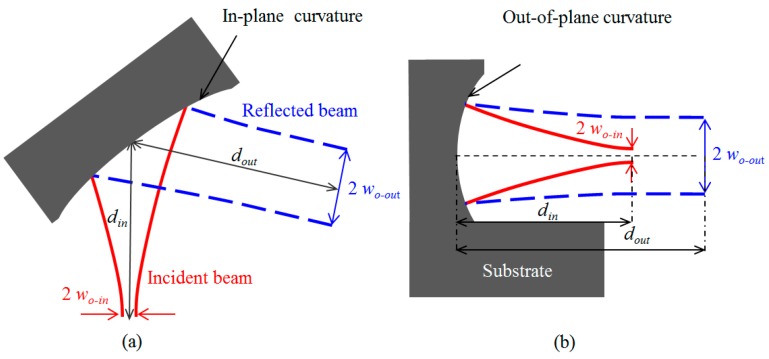
Three-dimensional curved micromirror used in beam collimation. (**a**) In-plane cross section; (**b**) out-of-plane cross section.

**Figure 3:**
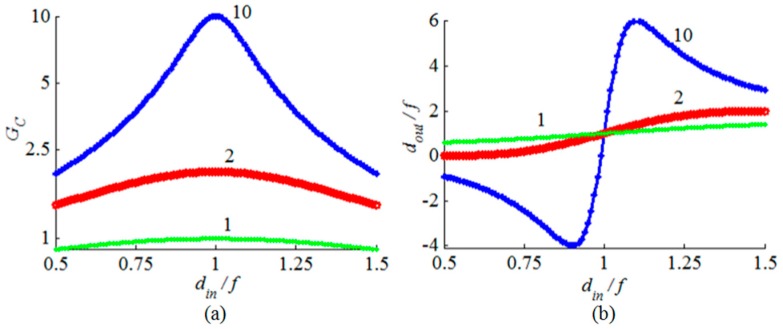
Dependence of the beam-waist ratio *G_c_* and the ratio *d_out_*/*f* on the ratio *d_in_*/*f* in (**a**) and (**b**), respectively, for different *f/z_o_* ratios.

Consider the incidence of a Gaussian beam on a curved micromirror as shown in Figure 2. The parameters of the reflected beam are related to the incident beam by:

(1) $$G_{c}=\frac{w_{out}}{w_{in}}=\frac{1}{\sqrt{{\left(1-d_{in}/f\right)}^{2}+z_{o}^{2}/f^{2}}}$$

(2) $$\frac{d_{out}}{f}=\frac{z_{o}^{2}/f^{2}-d_{in}/f(1-d_{in}/f)}{{\left(1-d_{in}/f\right)}^{2}+z_{o}^{2}/f^{2}}$$

where *w_in_* and *w_out_* are the min waist radii for the incident and reflected beams, respectively, *d_in_* and *d_out_* are the distances between the beam waist location and the mirror surface at the point of incidence for the incident and reflected beams, respectively, *f* is the focal length of the mirror and *z_o_* is the Rayleigh range of the incident beam. The beam-waist ratio *w_out_*/*w_in_* is denoted by *G_c_* and represents the collimation gain. The dependences of the beam-waist ratio and the ratio *d_out_*/*f* on the ratio *d_in_*/*f* for different ratios of *f*/*z_o_* are shown in Figure 3. The beam-waist ratio has a maximum value occurring when the input distance and the focal length are equal. The maximum beam-waist ratio is given by:

(3) $$G_{c}=\frac{f}{z_{o}}$$

The variation of the beam-waist ratio around *d_in_*/*f* = 1 is symmetric. The variation of the ratio *d_out_*/*f* possess odd symmetry around the point (*d_in_*/*f* = 1, *d_out_*/*f* = 1). The output beam waist location doesn’t change with the input beam Rayleigh range when the input beam waist is located at the focus of the mirror. Negative values of *d_out_*/*f* are obtained when *d_in_*/*f* < 1, which means the output beam waist is located virtually behind the mirror and the beam is diverging after reflection. The opposite case occurs when *d_in_*/*f* >1 and the beam is reflected in a converging state. The output beam waist may have its waist located just at the mirror surface for a single value of *d_in_*/*f* when *z_o_*/*f* = 2 and for two value of *d_in_*/*f* when *z_o_*/*f* = 0.5; one time for a very small value of *d_in_*/*f* and the second time for a *d_in_*/*f* that is slightly smaller than unity.

The microfabrication process tolerance may result in a variation of the curved micromirror radius of curvature, which affects the obtainable beam’s beam-waist ratio. The impact depends on the gain sensitivity to the curved surface focal length. The corresponding change is determined by:

(4) $$\begin{array}{ll} \Delta G_{c} & =\frac{\Delta f}{f}\frac{d_{in}/f(1-d_{in}/f)+{\left(z_{o}/f\right)}^{2}}{{\left[{\left(1-d_{in}/f\right)}^{2}+{\left(z_{o}/f\right)}^{2}\right]}^{3/2}}\\ & =\frac{\Delta f}{f}{\left(\frac{z_{o}}{f}\right)}^{-1},d_{in}/f\approx 1 \end{array}$$

**Figure 4:**
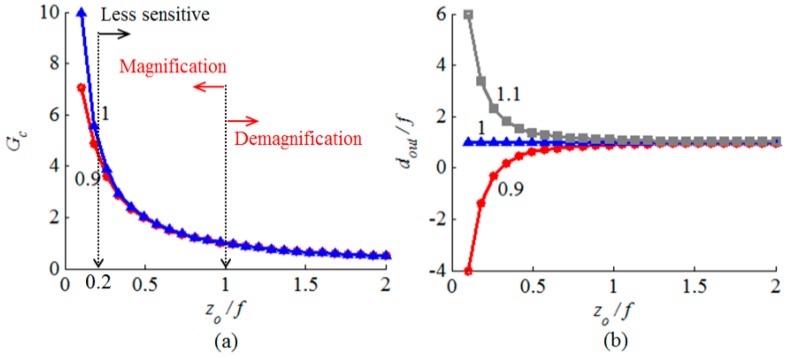
Dependence of the beam-waist ratio *Gc* and the ratio *d_in_*/*f* on the ratio *z_o_*/*f* in (**a**) and (**b**), respectively, for different *d_in_*/*f* ratios.

For a given percentage change in the focal length, the gain sensitivity becomes very high when the ratio *z_o_*/*f* is very small. As depicted in Figure 4a, the beam-waist ratio is less sensitive to the focal length variation when *z_o_*/*f* is larger than 0.2. The output beam waist location is, however, very sensitive to the variations as shown in Figure 4b. In the case of *z_o_*/*f* > 0.2, the fabrication tolerance impact on the output beam waist location can be compensated by active axial alignment.

The inclined incidence of the beam on the mirror in a tangential plane, while being normal to the sagittal plane, has the effect of splitting the focal length as well as the input ratio *d_in_*/*f* of the mirror each into two different values:

(5) $$f_{ip}=0.5R_{ip}\cos(\theta_{inc})$$

(6) $$f_{op}=0.5R_{op}/\cos(\theta_{inc})$$

(7) $${\left(\frac{d_{in}}{f}\right)}_{ip}=\frac{2d_{in}}{R_{ip}\cos(\theta_{inc})}$$

(8) $${\left(\frac{d_{in}}{f}\right)}_{op}=\frac{2d_{in}\cos(\theta_{inc})}{R_{op}}$$

where the subscripts “*ip*” and “*op*” are used for the in-plane and out-of-plane directions, respectively, and *R* is the radius of curvature of the mirror in the indicated plane. The inclined incidence has the effect of effectively increasing the out-of-plane focal length of the curved surface while at the same time decreasing its in-plane focal length, and therefore, a stigmatic inclined curved surface should have non-equal radii of curvature in the two orthogonal planes. As will be shown in the fabrication section, the out-of-plane plane radius of curvature can be limited to 100 μm. Fortunately, increasing angle of incidence compensates for this limit. For instance, focal length matching occurs at incidence angles *θ_inc_* = 0°, 45° and 60° for *R_op_*/*R_ip_* = 1, 0.5 and 0.25 respectively. Away from the stigmatic beam generation angle, the reflected beam exhibits an elliptical cross section as well different beam waist location in the two orthogonal planes. This can be of particular interest in beam shaping/matching applications.

## 3. Effect of Surface Roughness

The effect of the surface roughness expected from the micromachining of the 3-D curved surface on the collimated optical beam profile is investigated in this section. For this purpose, the overall phase transformation of the 3-D mirror is divided into the phase curvature responsible for the collimation of the beam, which is already considered in Section 2, and a random phase due to the surface roughness. The phase curvature corresponding to the curvature of the mirror surface is given by:

(9) $$\phi =\frac{2\pi }{\lambda }\frac{x^{2}+y^{2}}{2f}$$

where *f* is the equivalent focal length of the mirror. The random phase is given by:

(10) $$\phi_{n}=\frac{2\pi }{\lambda }z_{n}$$

where *z_n_* = *f(x,y)* is the random height variation of the surface due to the surface roughness. In our analysis, *f(x,y)* is assumed a random rough surface that has a Gaussian height distribution function and Gaussian autocovariance functions (in both *x*- and *y*-direction). The surface is assumed to have an RMS height *σ_rms_* and assumed to be isotropic in the sense that the correlation length *L_c_* in the *x*- and *y*-direction are assumed equal.

The simulation procedure is carried out using the Fourier optics approach as follows [32]. The field at the mirror surface, denoted by *E_in_*(*x*,*y*,*d_in_*), is multiplied by the phase transformation function and the new output field is denoted by *E_o_*(*x*,*y*,*d_in_*) :

(11) $$E_{o}(x,y,d_{in})=E_{i}(x,y,d_{in})\exp(-j\phi_{n}-j\phi )$$

A fast Fourier transform (FFT) is applied to get this output field in the spatial frequency domain:

(12) $$G_{o}(f_{x},f_{y},d_{in})=FFT\left\{E_{o}(x,y,d_{in})\right\}$$

The field is propagated a distance *d_out_* by phase multiplication in the spatial frequency domain:

(13) $$G_{o}(f_{x},f_{y},d_{out})=G_{o}(f_{x},f_{y},d_{in})\exp(-jk_{z}d_{out})$$

where *k_z_* is the axial components of the wave vector. Finally, the output field profile after propagating the distance *d_out_* is obtained by inverse Fourier transform:

(14) $$E_{o}(x,y,d_{out})=IFFT\left\{G_{o}(f_{x},f_{y},d_{out})\right\}$$

A simulation study was carried out to analyze the effect of the surface roughness of the etched mirror on the collimated beam. The effect is evaluated by calculating the coupling efficiency (overlap integral) between the resulting and the ideal beam. The radius of curvature of the mirror in the in-plane direction is assumed 300 µm, while the out-of-plane radius of curvature is 150 µm, similar to the value obtained practically as will be shown in the next section. The incident beam has a minimum waist radius of 5 µm, a wavelength of 1550 nm and located at the focal plane of the mirror in a 45-degree incidence orientation. The RMS roughness *σ_rms_* is assumed in the range of 0 to *λ*/10. Three values of the correlation were assumed: 5*λ*, *10λ* and *20λ.*

**Figure 5:**
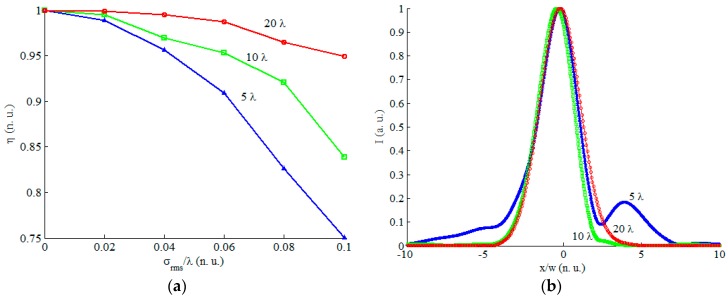
Effect of surface roughness on coupling efficiency and collimated beam profile. (**a**) Coupling efficiency versus RMS roughness normalized to the wavelength at different roughness correlation lengths; (**b**) collimated beam profile versus the transverse dimension normalized to the ideal beam waist radius.

The resulting coupling efficiency is depicted in Figure 5a. Since the roughness generation is a stochastic process, the simulation was repeated 20 times for each point and the average was taken. The coupling efficiency decreases with the increase of the RMS value of the roughness, as expected. It reaches about 75% for the case of *L_c_* = 10*λ* and *σ_rms_* = *λ*/10. If we would like to maintain at least 95% of the coupling efficiency, then *σ_rms_* should be less than 0.04*λ*, 0.06*λ* and 0.1*λ* for *L_c_* = 5*λ*, 10*λ* and *L_c_* = 20*λ*, respectively. Example resulting beam profiles for the case of *σ_rms_* = 0.1*λ* are shown in Figure 5b. The *x*-axis is normalized to the waist of the resulting beam profile in case of The loss in efficiency is resulting from the asymmetry in the beam profile in addition to the widening of the profiles out of the ±4*w* limit due to the surface roughness.

## 4. Silicon Micromirror Fabrication

**Figure 6:**
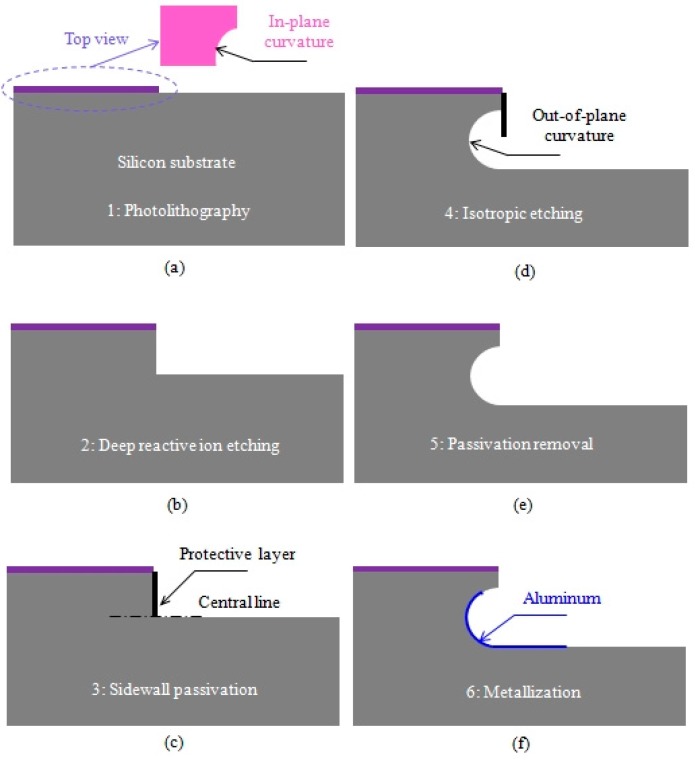
The fabrication steps of the collimating 3-D curved micromirror. (**a**) Photolithography, (**b**) deep reactive ion etching, (**c**) sidewall passivation, (**d**) isotropic etching, (**e**) passivation removal, and (**f**) metallization.

**Figure 7:**
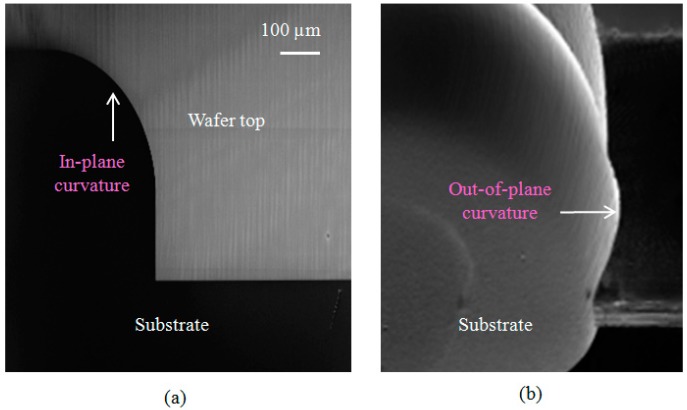
Scanning electron microscope (SEM) images of the fabricated micromirror. (**a**) Top view where the in-plane curvature is emphasized; (**b**) tilted view where the out-of-plane curvature is emphasized.

The optical axis of the target 3-D curved micromirror lies in-plane with respect to the wafer substrate to collimate the optical beam generated from single-mode optical fibers located horizontally on the wafer substrate or any other light source integrated in the system. It enables the use of the fiber-mirror configuration to replace the lensed fiber as previously shown in Figure 1d. The fabrication of the micromirror was carried out into six main steps [33]. First the definition of the in-plane profile of the micromirror with a 300-μm radius of curvature was performed using standard photolithography (see top view in Figure 6a). The lithographic process ends with a patterned SiO_2_ mask layer for the following etching. Second, anisotropic deep reactive ion etching of the silicon was carried out, ending with a deeply etched cylindrical surface as shown in Figure 6b [34]. By this anisotropic etching step, the central line of the out-of-plane curvature (principal axis) is defined. The axis depth with respect to the wafer top surface was chosen to be large enough that optical fiber can be inserted and aligned with micromirror. Then, side wall protection was carried out using a Teflon-like layer to prevent sidewall etching from top and ensure the following isotropic etching starts at the mirror principal axis as shown in Figure 6c. The protection step was followed by a long isotropic etching step using SF_6_ plasma to achieve the desired out-of-plane profile of the micromirror as shown in Figure 6d, in a similar way to that used to fabricate micro fluidic channels reported in [35]. The out-of-plane radius of curvature of the micromirror surface is about 150 μm. Achieving larger radii of curvatures requires deeper etching, which may result in a fragile wafer. The protective layer was removed in the fifth step as shown in Figure 6e using a high-temperature oxygen plasma ashing process. As will be shown below, the resulting surface roughness was about 22 nm RMS. Therefore, the surface was post-processed for optical quality requirement by smoothing and Aluminum metallization as shown in Figure 6f. Top and tilted views of the fabricated micromirror after step 5 are shown in Figure 7a,b, recorded using a scanning electron microscope (SEM). 

**Figure 8:**
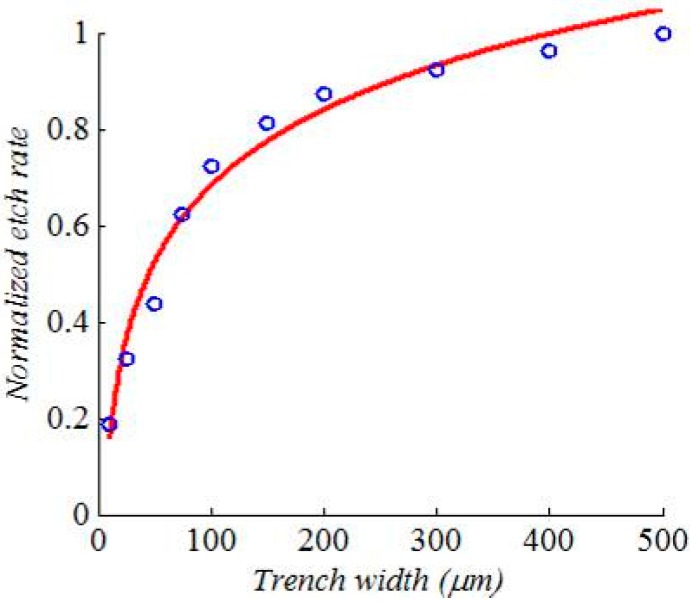
Normalized isotropic etching rate versus the etched trench opening width while. The trench length is 300 μm. The measured data (in markers) is fitted to a logarithmic function (in line).

**Figure 9:**
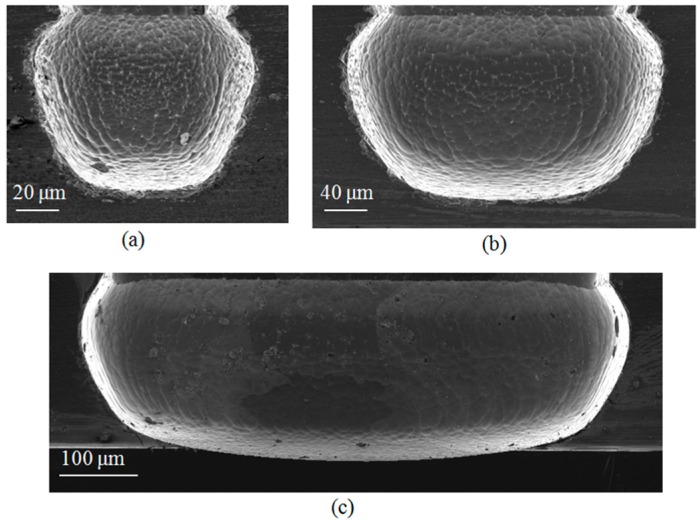
SEM images showing the roughness of the isotropically-etched trenches. The opening widths are 75 μm in (**a**); 150 μm in (**b**) and 500 μm in (**c**).

**Figure 10:**
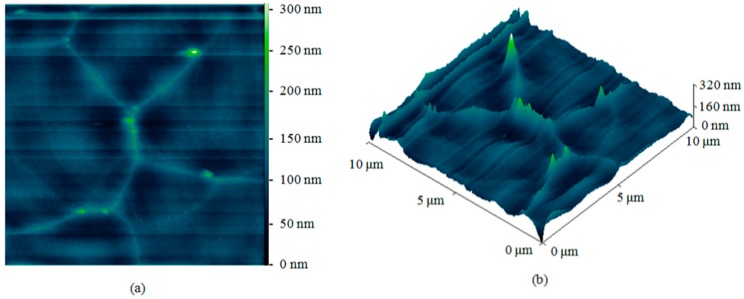
The isotropic etching roughness measured in a 500 μm trench using the atomic force microscope (AFM). A top view of the measured surface is shown in (**a**) while a tilted 3-D view is shown in (**b**).

More than one effect was encountered regarding the isotropic etching of silicon using SF_6_. First, a significant dependence of the etch rate on the trench width was observed, as shown in Figure 8. The etch rate is normalized with respect to the etch rate of the largest trench width. The data markers represent the measured normalized data while the solid line is a logarithmic fitting. This kind of logarithmic behavior is well-known for a diffusion-limited etching process [14]. The etch rate for a 10 μm trench width is about one fifth the rate for a 500 μm trench width. The second observation is the correlation between the mask opening width and the isotropic etching roughness as shown in Figure 9. The smaller the mask opening is, the higher the roughness. Considerable roughness can be observed in the smallest opening by inspecting the SEM images with the naked eye, while the roughness in the largest opening is much less, but still observable. The atomic force microscope (AFM) was used in order to get a quantitative measurement for the roughness of the largest opening. The top and 3-D tilted views of the surface topology, obtained using the AFM on an area of 10 μm by 10 μm, are shown in Figure 10a,b respectively. The measured roughness has a peak of 319 nm, an average of 16 nm and an RMS 22 nm. The lag effect as well as the surface roughness of the isotropic etching roughness can be interpreted knowing that a diffusion process governs the transport of the etching radicals from the plasma, where it is created, to the substrate, where chemical etching occurs. Due to this diffusion process, a lower amount of etchants is received in thinner trenches. This directly relates to the lag effect. At the same time, when the amount of etchants is not enough, a rough surface results from the etching process because the surface is not overwhelmed by the etchants.

## 5. Measurement Results and Discussion

**Figure 11:**
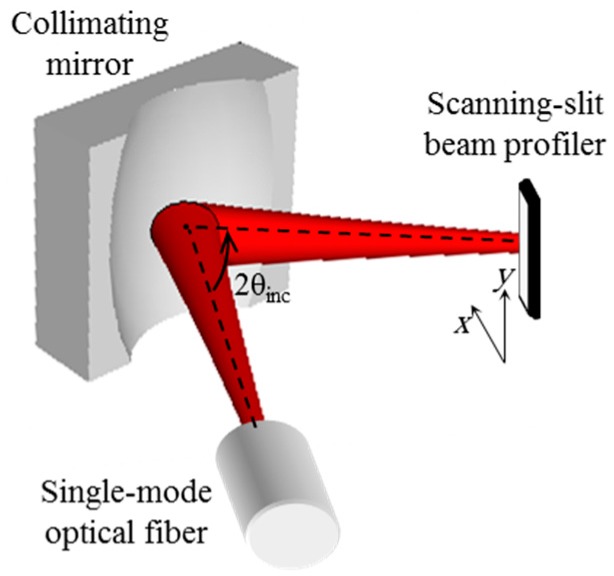
Measurement setup of the reflected beam from the fabricated mirror.

In this section, the manufactured 3-D curved micromirror is utilized for collimating the output beam of single-mode fibers and propagation loss reduction thereof. Consider the arrangement shown in Figure 11.

A single-mode optical fiber is inserted on the silicon substrate such that its optical axis is parallel to the silicon substrate and tilted with respect to the mirror principal axis. For the sake of optical spot characterization, the reflected beam is captured in the far field on a scanning-slit beam profiler. The observed beam ellipticity, defined by the ratio of the spot size in the in-plane direction to the out-of-plane direction, is adjusted to be close to unity (about 1.05) by letting the incidence angle of the beam on the mirror be about 45°. The axial distance between the optical fiber and the mirror was adjusted such that the fiber tip is located at the micromirror focal plane by minimizing the observed output beam diameter at the far field. The collimated output beam spot diameter was measured at different locations away from the micromirror and compared to the measurements of the optical fiber output beam without using the micromirror. 

**Figure 12:**
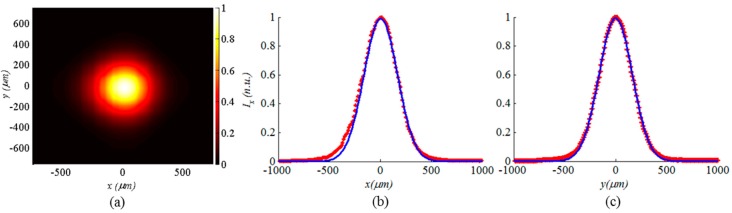
Measured spot profile: (**a**) contour plot; (**b**) in-plane beam profile (markers) fitted to a Gaussian profile (line), and (**c**) out-of-plane beam profile (markers) fitted to a Gaussian profile (line).

In the case of using a standard single-mode fiber with a core radius of 4.5 μm fed from 1550 nm laser source, a reduction in the divergence angle of the beam by a factor of 2 was achieved by the micromirror. The output beam has a minimum waist radius of about 10 μm, which is a typical value for many optical MEMS applications. A typical captured beam profile at one location *d* is shown in Figure 12a. The profile was fitted to a Gaussian profile with average root mean square errors smaller than 1% and 1.5% in the *x*- and *y*- directions respectively as shown in Figure 12b,c. This is an indication of the good performance offered by the fabricated micromirror, using the presented method, in terms of its phase front transformation function. This experiment was repeated with a special single-mode fiber with a core radius of 2 μm working at a 675 nm wavelength. The special fiber is positioned at the same location used for the standard one because of the constant focal length of the mirror independent of the wavelength value. A reduction in the divergence angle of the beam by a factor of 4.25 was achieved. The resulting output beam has a minimum waist radius of about 10 μm as well. This visible beam will be used hereinafter for evaluating the propagation loss reduction offered by the micromirror.

**Figure 13:**
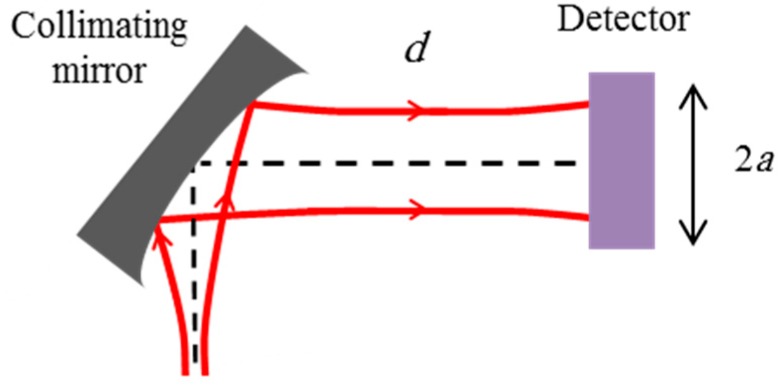
Measurement setup of the power on a detector with aperture radius *a*.

The collimation of the beam by the micromirror was also evaluated by measuring the detected power in free space with a limited-aperture detector as shown in Figure 13. 

Theoretically, the transmitted power in terms of the system aperture radius *a* and the beam spot radius at the detector is given by [36]:

(15) $$P=1-\exp\left(-2\frac{a^{2}}{w^{2}}\right)$$

**Figure 14:**
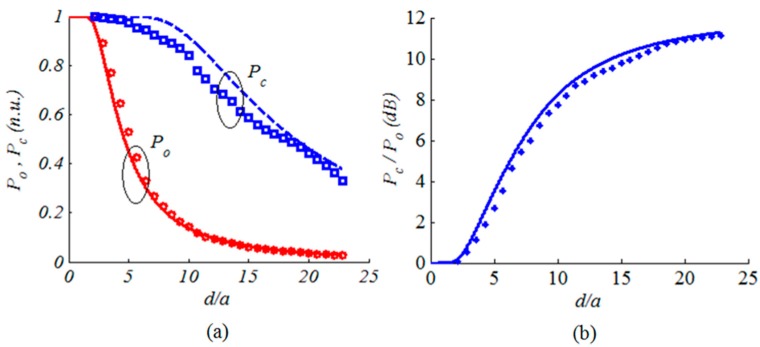
(**a**) The normalized power collected by the detector; (**b**) diffraction loss reduction in dB using the collimating micromirror. The measured data is given in markers when the theoretical one is given in lines.

The power collected by a detector with 3.5 mm aperture radius is shown in Figure 14a. The power was measured at different distance *d* in the far field away from the beam waist. The measurements were carried out one time for the collimated beam by the micromirror, denoted by *P_c_*, and another time for beam originally emitted by the single-mode fiber, denoted by *P_o_*. The experimental data are depicted using markers while the theoretical data are depicted using lines. The power is normalized with respect to the initially maximum power. The measured power clearly starts to fall when the beam diameter starts to exceed the detector aperture as given by Equation (9). The micromirror significantly reduces the propagation losses with respect to the original fiber output. The detected power from the micromirror has a slower roll-off and drops to half its maximum value 25 cm far from the micromirror compared to less than 8 cm without using the micromirror. The ratio between the two detected powers is depicted in Figure 14b, where the improvement reaches about 11–12 dB. Indeed, in the far field, the ratio between the detected powers is given by:

(16) $$G_{p}=\frac{P_{c}}{P_{o}}=\frac{1-\exp\left(-2\frac{a^{2}}{\theta_{div-c}^{2}d^{2}}\right)}{1-\exp\left(-2\frac{a^{2}}{\theta_{div-o}^{2}d^{2}}\right)}$$

where the beam spot radius in the far field was replaced by *wd*/*z_o_* = *d*/*θ_div_*. The maximum improvement is achieved when the spot radius becomes much larger than the detector aperture. In this case, Taylor expansion of the exponential terms can be applied to second order and Equation (16) becomes:

(17) $$G_{p-\max}=\frac{\theta_{div-c}^{2}}{\theta_{div-o}^{2}}=G_{c}^{2}$$

The maximum power gain due to the usage of the collimating mirror is given by the beam-waist ratio squared. For the fabricated micromirror and using the single-mode fiber at 675 nm, the power gain is *G_p_* = (4.25)^2^ = 18 that is about 12.5 dB, in good agreement with the measured data. This value is independent of the specific sizes of the beam spot and the detector aperture, as long as significant truncation loss is encountered.

## 6. Conclusions

Optical beam collimation was analyzed and successfully carried out using a micro-reflector with a three-dimensional curved surface. The surface was etched in silicon by a technique combining deep reactive ion etching and isotropic etching technologies. The produced surface is astigmatic with an out-of-plane radius of curvature that is about half the in-plane radius of curvature. Having the incident beam in-plane and inclined by 45° with respect to the principal axis, the reflected beam is kept stigmatic with about a 4.25-fold reduction in the beam expansion angle in free space and about 12-dB reduction in propagation losses. The fibre–mirror configuration may serve as a potential replacement for the lensed fibers widely used in the MOEMS system. This replacement has the advantage of producing monolithically integrated systems with a wider-band spectral response.

## Abbreviations

The following abbreviations are used in this manuscript:

RMS	Root mean square
MOEMS	Micro-opto-electro-mechanical systems
RIE	Reactive ion etching
PR	Photo resist
3-D	Three-dimensional
OB	Optical bench
SEM	Scanning electron microscope
AFM	Atomic force microscope
